# Vulvar basal cell carcinoma: A case report and literature review

**DOI:** 10.1016/j.ijscr.2023.108382

**Published:** 2023-06-02

**Authors:** Soheila Aminimoghadam, Zahra Marzban, Marjan Ghaemi, Sepideh Azizi

**Affiliations:** a Department of Obstetrics and Gynecology, Firoozgar Hospital, School of Medicine, Iran University of Medical Science, Tehran, Iran; b Vali-E-Asr Reproductive Health Research Center, Family Health Research Institute, Tehran University of Medical Sciences, Tehran, Iran; c Shahid Akbarabadi Clinical Research Center, Iran University of Medical Science, Tehran, Iran

**Keywords:** Carcinoma, Basal cell carcinoma

## Abstract

**Introduction and importance:**

Basal cell carcinoma (BCC), is the most prevalent skin cancer with favorable prognosis, and lymphatic or hematogenous metastases are quite rare. Here we report an uncommon case of vulvar BCC.

**Case presentation:**

A 68-year-old woman with 5-year history of an asymptomatic lump on the right side of the vulva. The patient complained of progressive itching, pain, and color changes to brown for 3 months before visiting the clinic. Excisional biopsy revealed the lesion to be a BCC. After thorough negative metastasis work up the patient underwent a bilateral inguinofemoral lymphadenectomy and a wide local excision with a one-centimeter margin and final histology report confirmed the diagnosis of BCC. (HPV DNA and viral markers were negative, the bimanual exam was normal, both colposcopy and vaginoscopy were normal. There was no acetowhite lesion).

**Clinical discussion:**

To avoid delayed diagnosis, any persistent lesion even asymptomatic, in the vulvar region, especially when pigmented, irritating, or expanding in size, should be biopsied and investigated histologically, regardless of the patient's age.

**Conclusion:**

Any persistent lesion in the vulvar region, especially when pigmented, irritating, or expanding in size, should be biopsied and investigated histologically, regardless of the patient's age.

## Highlights

• Vulvar BCC exhibits a more aggressive clinical behavior compared with BCC arising in sun exposed areas.
• Persistent vulvar lesions, especially pigmented, irritating, or expanding lesions, should be biopsied and histologically investigated to avoid delayed cancer diagnosis.
• Vulvar BCC Prognosis is favorable if managed appropriately.

## 1 Introduction

Basal cell carcinoma (BCC), the most prevalent form of skin cancer, is highly treatable. This tumor has a favorable prognosis, and lymphatic or hematogenous metastases are quite rare [1]. Age and sun exposure are both significantly linked with the incidence of BCC. The majority of tumors are found in the head and neck. BCC can also occur in areas of the body that are primarily shielded from the sun, such as the perineum, axillae, buttocks, and groin [2].

Vulvar BCC is uncommon and accounts for 2 to 4 % of all vulvar cancers and <1 % of all BCCs. The majority of affected women are white, and their average age is 70 [[3], [4], [5]]. In vulvar area BCC is characterized by poor pigmentation and a clinical appearance often mimicking other dermatological pathologies like eczema or psoriasis. The dominant symptom is itching, bleeding, ulceration, pain and discomfort [6].

Vulvar BCC exhibits a more aggressive clinical behavior compared with BCC arising in sun exposed areas. Higher frequency of clinical recurrences and regional or distance metastasis have been reported for vulvar BCC [5,7]. Biopsy and histologic study are the most definitive method for confirming BCC. Without histopathologic confirmation, clinical suspicion alone is insufficient to proceed with treatment. Various biopsy techniques may be used, including excisional, incisional, shave, and punch biopsies. Punch and shave biopsies have been shown to have similar diagnostic accuracy.

Treatment options for vulvar BCC include radical and simple vulvectomy, wide local excision, simple surgical (local) excision, and Mohs micrographic surgery (MMS) [8]. Herein, we report a case of vulvar BCC who has presented with chronic itching. This case presentation is written according to SCARE 2020 checklist [9].

## 2 Case report

A 68-year-old woman presented to our clinic with a history of a asymptomatic lump on the right side of the vulva. She had the lesion for five years but never sought professional advice. The patient complained of progressive itching, pain, and color changes to brown for 3 months before visiting the clinic.

The assessment revealed a 14–15 mm diameter brown pigmented plaque without ulceration. The patient had no prior history of radiation therapy, tanning and family history of skin cancer and she was otherwise healthy. Pelvic exam revealed no abnormality. An excisional biopsy was preformed and histopathology report revealed a superficial BCC. Following the histology findings, the patient was referred to a gyneco-oncologist and underwent a complete evaluation.

HPV DNA and viral markers were negative, the bimanual exam was normal, both colposcopy and vaginoscopy were normal. There was no acetowhite lesion. Transvaginal sonography revealed normal uterus and adnexa without pelvic lymphadenopathy. The non-contrast abdominopelvic CT scan revealed no lymphadenopathy, and the liver, spleen, and other abdominopelvic organs were all normal.

**Fig. 1:**
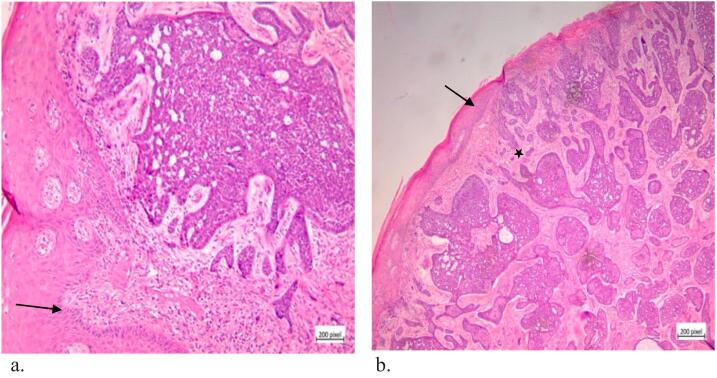
a, b: BCC tumor. a: The black arrow shows palisade around tumor. In histopathology a palisade is a single layer of relatively long cells, arranged loosely perpendicular to a surface and parallel to each other. b: The black arrow shows the outer layer (epidermis). The black star is proliferation of nests of small basal cells with high nuclear to cytoplasmic ratio.

The treatment team including oncologist and gyneco-oncologist planned a surgery including bilateral inguinofemoral lymphadenectomy and a wide local excision with a one-centimeter margin in depth and width. The pathology report was BCC (Fig. 1a, b).

In follow-up the patient is coming to the clinic every three months. We did physical exam, bimanual exam, speculum exam and examine lymphadenopathy. There was no new detection to do extra imaging. She had not any complaints for 2 years.

## 3 Discussion

Vulvar BCC is a rare condition with unknown etiology [10]. Symptoms persist for an extended period of time. Factors associated with an increased risk of BCC include mutation in tumor suppressor and regulatory genes. The role of the immune system in the pathogenesis of BCC is unclear. Immunosuppressed patients with lymphoma or leukemia and patients who have under gone transplants show a marked increase in BCC [10]. Indeed, BCC is associated with damage caused by exposure to sunlight and radiation, arsenic ingestion, immunosuppression and inherited conditions, such as xeroderma pigmentosum and nevoid BCC syndrome [11,12].

As the pathogenesis of BCC in other anatomical sites most commonly correlates with exposure to ultra violet (UV) light, but there is no clear explanation for the occurrence of this tumor in the vulva [13].

Vulvar BCC commonly presents after seventh decades of life. Concerning socioeconomic factors, it seems low income and limited access to healthcare can contribute to the development of vulvar BCC. Women who live in poverty may be less likely to receive preventive screening for vulvar cancer, leading to an increased risk of advanced disease at presentation. The diagnosis of vulvar BCC is often delayed because of slow progression and atypical presentation in the vulvar area [6]. Patient reluctance to seek medical attention is the main reason behind the delayed diagnosis. Etiological risk factors include: chronic exposure to arsenic, basal cell nevus syndrome and chronic irritation. BCC in sun protected skin are thought to be due to chronic irritation and inflammation secondary to the lesions like lichen sclerosis [2].

Genetic defects have been shown to predispose the development of BCC. For example, the protein patched homolog 1(PTCH) mutation found in basal cell nevus syndrome patients substantially increase susceptibility to BCC [2].

Although BCCs are generally slow growing tumors, they are locally invasive and destructive and carry the potential to recurrence and metastasis [13]. Aside from the case presented in this paper, which had the lesion for five years, her workup revealed no evidence of metastases. Metastatic vulvar BCC frequently involves inguinal lymph nodes, bone, lung and skin. In addition to the vulvar location of BCC, aggressive histologic subtypes (morpheaform, infiltrating, and basosquamous), large tumor size (>2 cm), and lack of UV exposure are also associated with an increased risk of metastasis [13].

Histopathologically, vulvar BCC resembles BCC found in other anatomic sites. Certain histologic forms, such as basosquamous carcinoma, adenocystic, and infiltrative, tend to be more aggressive, which may explain recurrence cases [2].

M Reyes et al. in a case series reported that all four cases of vulvar BCC were positive for ck5/6, βcatenin and p63 which are exposed in most BCC independently of their localization. Vulvar BCC is a non-Human Papilloma Virus (HPV) related neoplasm that seems to be commonly associated with chronic vulvar irritation. HPV DNA has been detected in BCC arising from sites other than genitalia, not all cases of genital BCC have revealed the presence of HPV DNA. A significant relationship between HPV and BCC remains to be established [7]. Abnormal β catenin staining is a frequent in several neoplastic vulvar lesions such as Squamous Cell Carcinoma (SCC) and Extra mammary Paget disease and has postulated as a useful marker to differentiate neoplastic from non-neoplastic vulvar lesion. Expression of p63 pro (a protein required for cutaneous development and frequently over expressed in SCC and BCC) was detected in vulvar BCC [7].

Infiltrative BCC has been demonstrated in patients with Human Immunodeficiency Virus (HIV) infection. In a series of BCC cases 35/7 % of 28 patients with vulvar BCC were noted to have another malignancy, but this frequency is probably not greater than what might be expected in a similar group of elderly women with a mean age of 73 years [10].

Options for managing vulvar BCCs include radical and simple vulvectomy, wide local excision, simple surgical (local) excision, and Mohs micrographic surgery (MMS). The similar case reports are presented in Table 1.

**Table 1:** The similar cases of vulvar BCC and their management.

Clinical findings	Surgical procedure	Follow up	References
62 y/o, vulvar lesion, indurated, smooth, ulcerated, shiny plaque, 13 ∗ 9 mm, hairy area of the vulva	Wide local excision	Follow up with an immunohistochemical pattern, high expression of CK7	[7]
86 y/o, foul-smelling bloody discharge for 7 months, hip pain, weight loss, femoral bone metastasis	Bilateral radical resection of the vulva, the mons anteriorly and the perineum posteriorly, 2 cm margin	Palliative radiation therapy died peacefully	[14]
66 y/o, solitary vulvar plaque lesion, had bled on sometimes, 1.5 cm, posterior third of labia major, near the fourchette	Local excision	It recurred after 3 years of follow-up. Wide local excision is done, then.	[7]
93 y/o, vulvar irritation, focally ulcerated indurated plaque, right vulva, 5 cm	Wide local excision	49 months follow up	[15]
65 y/o, non-pigmented nodule with bleeding 2 cm	Local excision	30 months	[7]
55 y/o, ulcerated nodule, 17 ∗ 12 mm, right vulva	Wide local excision	102 months follow up	[15]

The standard of care for treatment of BCC is wide local excision with pathologically proven clear margins of approximately 1 cm. Postoperative radiation dose not appear to affect recurrence rate or overall survival and is associated with unpleasant side effects and poor cosmetic result. Systematic chemotherapy is not indicated in the treatment of localized BCC and has been used in metastatic disease. Surgical excision remains the accepted treatment for primary and recurrence localized vulvar BCC [10]. Prognosis is favorable if managed appropriately.

## 4 Conclusion

In conclusion, vulvar BCC remains to be an uncommon vulvar tumor with a great prognosis if managed appropriately. Any persistent lesion in the vulvar region, especially when pigmented, irritating, or expanding in size, should be biopsied and investigated histologically, regardless of the patient's age.

## Consent for publication

The written informed consent was obtained from the patient for publication of this case report and accompanying images. A copy of the written consent is available for review by the Editor-in-Chief of this journal on request.

## Ethical approval

Ethical approval for this study (approval code: 566233) was provided by the Ethical Committee of Iran University of Medical Sciences, Tehran, Iran on 26 November 2022.

## Funding

Not applicable.

## Author contribution

Sepideh azizi: manuscript writing

Marjan Ghaemi: manuscript editing

Soheila Aminimoghadam: conceptualization

Zahra Marzban: figure preparation

## Guarantor

Sepideh Azizi.

## Provenance and peer review

Not commissioned, externally peer reviewed.

## Declaration of competing interest

Not applicable.
